# Bioactivity of an organic farming aid with possible fungistatic properties against some oil palm seedling foliar pathogens

**DOI:** 10.1038/s41598-023-27972-y

**Published:** 2023-01-23

**Authors:** Joshua Obeng, Daniel Agyei-Dwarko, Peter Teinor, Isaac Danso, Hanif Lutuf, Emmanuellah Lekete-Lawson, Fred Kormla Ablormeti, Mary Akpe Eddy-Doh

**Affiliations:** 1 Council for Scientific and Industrial Research – Oil Palm Research Institute, P. O. Box 74, Kade, Ghana; 2 HJA Africa Limited, Accra, Ghana

**Keywords:** Biological techniques, Microbiology, Plant sciences

## Abstract

Synthetic fungicides are necessary evil in crop production, their usage cannot be neglected or abandoned but must be alternated/supplemented with other control measures such as cultural, host resistance and biocontrol methods to reduce their detrimental effect on the environment and living organisms. A bioproduct (wood vinegar) was evaluated against oil palm seedling pathogens at CSIR—Oil Palm Research Institute, Kusi at different concentrations and compared with an inorganic fungicide at the manufacturer’s recommended dosage. Disease pathogens were isolated from collected diseased leaf samples and pure cultures were established on cPDA. PDA was amended with wood vinegar ranging from 0 to 3.35% and 0.1%v/v of carbendazim as a positive control. Daily colony growth was measured in two diagonal lengths and averages of day 6 and day 7 were used to calculate the inhibition percentage for both pathogens. 11 mm/day was the lowest average growth rate recorded for 2.68% v/v of wood vinegar and 14.17 mm/day on control plate of *Curvularia* species. There was no significant difference between 0.1%v/v carbendazim, 2.68 and 3.35% v/v against *Curvularia* species whilst significantly, there was difference between 0.1%v/v carbendazim and 2.68 and 3.35%v/v of wood vinegar against *Pestalotiopsis* species.

## Introduction

Oil palm (*Elaeis guineensis*) originated from Africa and is one of the significant cash crops in the genus *Elaeis* in terms of production and economic yields^1,2^. The usual practice in the cultivation of oil palm is to raise nursery where germinated seeds are sown in poly bags filled with topsoil. The seedlings are maintained in the bags from six to twelve months before being transplanted on the field^3^.

However, seedling growth and development is usually affected noticeably by soil fertility, shade regime, moisture supply, weed competition and most importantly, pest and disease incidence. Fungi are the major disease causal organisms on oil palm seedlings, suppressing their growth and subsequently reducing yield^4^. More than sixty diseases and disorders have been reported around the world^5,6^. In Ghana, predominantly, spear rot, anthracnose, Curvularia leaf spot, Cercospora leaf spot and Pestalotiopsis leaf spot are some of the diseases reported in established nurseries^2^. Over the years, synthetic or inorganic fungicides have been and are being used to manage these fungal diseases in the country. However, there are noted resistance of *Aspergillus*, *Candida* spp and *Curvularia* spp to azoles especially fluconazole, voriconazole and also, echinocandins^7,8^. Several countries in Europe, South America, Asia, Central America and Africa have also noticed this antifungal resistance clinically^9,10^. Snelders et al.^9^ reported that fungicides usage in agriculture by applicators, exposed to these chemicals for treating and preventing fungal diseases in crops can contribute to resistance in people exposed to these fungicides. Resistance can also develop overtime when fungi are exposed to antifungal drugs or chemicals for prolonged period^11,12^. *Botrytis cinerea* has been reported by Hahn^13^ to be resistant against several inorganic fungicides due to how they were used. Organic Farming Aid (OFA) wood vinegar® contains Pyroligneous acid (PA) which is a byproduct of bio-oil obtained by pyrolysis of the wood. It is produced and expected to be marketed in Ghana by HJA Africa in Accra. The use of wood vinegar has/is been or being extensively used in Asia specifically Japan for some time now but this OFA (wood vinegar) is the first time we are evaluating such product against oil palm foliar pathogens to determine its efficacy.

Due to antifungal resistance and environmental health related issues associated with synthetic pesticide, organic products with fungistatic or fungicidal effect are worth trying. The objective of this study is to assess efficacy of different concentrations of foliar fertilizer (Organic Farming Aid-Wood vinegar) against two nursery disease pathogens and the fungistatic activities of the organic product on different developmental stages of the pathogens.

## Results

### Inhibitory effect of wood vinegar on mycelial growth

**Figure 1:**
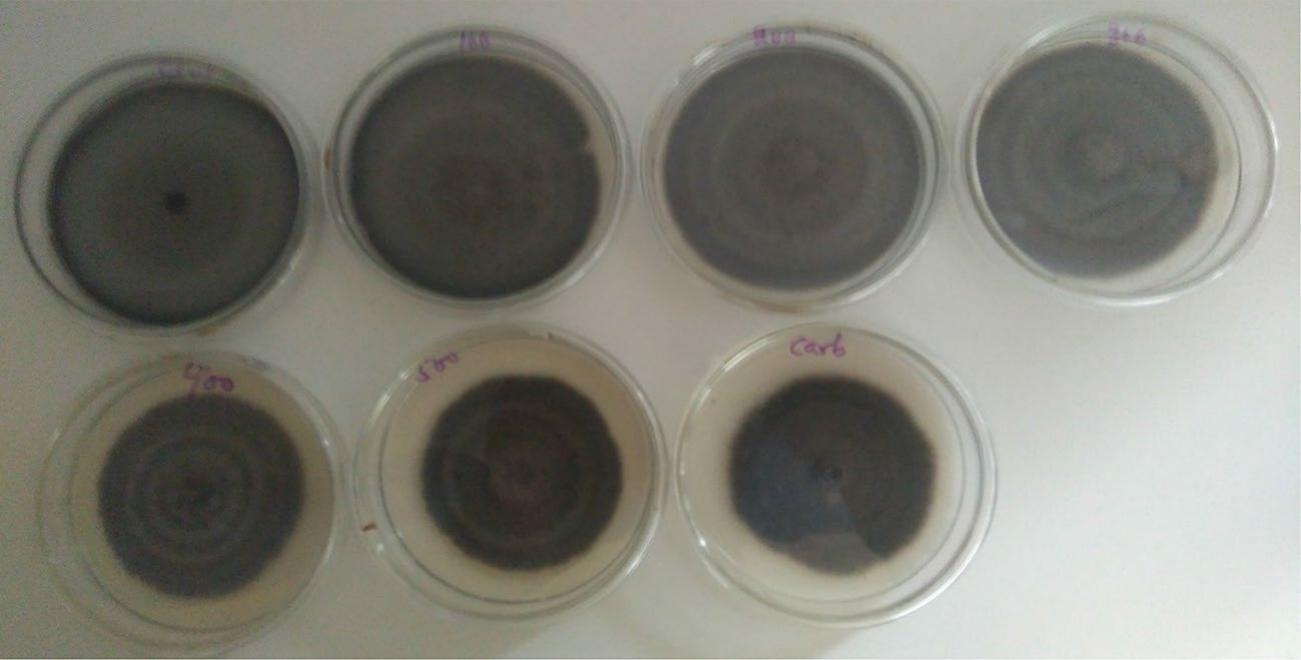
*Curvularia* species growing on PDA amended with different concentrations of wood vinegar and carbendazim at day 6. (Top row L–R; Control, 0.67, 1.34, 2.01 (%), bottom row L–R; 2.68, 3.35 and Carbendazim 0.1%).

**Figure 2:**
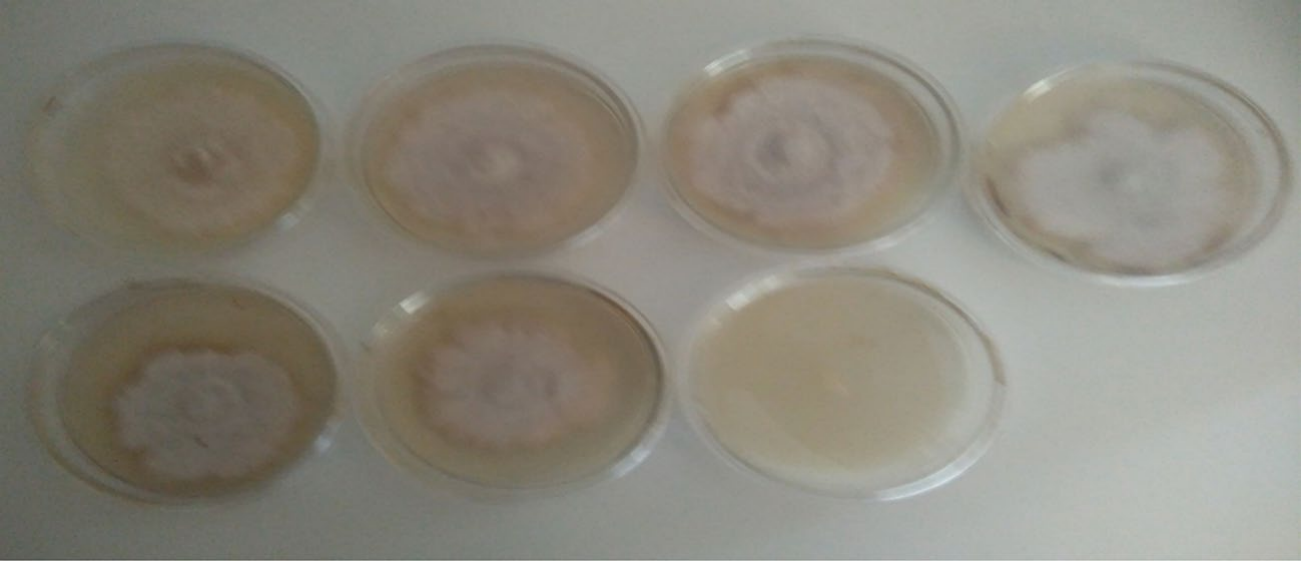
*Pestalotiopsis* species growing on PDA amended with different concentrations of wood vinegar and carbendazim at day 7. (Top row L–R; Control, 0.67, 1.34, 2.01 (%), bottom row L–R; 2.68, 3.35 and Carbendazim 0.1%).

In-vitro evaluation of wood vinegar on mycelium growth of *Curvularia* and *Pestalotiopsis* species at different concentrations was promising when colony diameter was measured. 23.35 and 28.56% were the highest inhibition percentage recorded for *Curvularia* and *Pestalotiopsis* species, respectively. The highest concentration (3.35% v/v) recorded the highest inhibition percentage of 28.56% which was not statistically different from the concentration (2.68% v/v) for *Pestalotiopsis* species whilst both concentrations’ inhibition percentages were significantly different from carbendazim at 0.1% v/v, which outperformed the best performing concentrates of the wood vinegar. The two highest concentrations of the wood vinegar (2.68 and 3.35% v/v) were not significantly different from carbendazim at 0.1% v/v when tested against *Curvularia* species (Table 1, Figs. 1, 2). However, a concentration was considered to be inhibitory, if inhibition percentage was ≥ 20%.

**Table 1:** Percentage inhibition of pathogens at different concentrations of the Organic Farming Aid (Wood vinegar).

Treatments (% v/v)	Percent inhibition^a^			
	Pathogens			
	*Pestalotiopsis* species		*Curvularia* species	
	Day 7^c^	Day 7^d^	Day 6^c^	Day 6^d^
0 (Control)	0.00a	0.00a	0.00a	0.00a
0.67	0.59b	3.08a	0.74b	5.06b
1.34	0.63b	3.46a	0.98bc	8.71bc
2.01	1.11c	12.25b	1.10cd	11.65c
2.68	1.36d	22.23c	1.37e	22.35d
3.35	1.47d	28.56d	1.38e	23.35d
Carbendazim (0.1)^b^	2.00e	100.00e	1.31de	19.41d
CV	3.2	3.5	2.8	2.6

^a^Percentage inhibition was calculated from mean values of colony diameter obtained from the various concentrations.

^b^Carbendazim was a positive control and the manufacturer’s recommended rate was duly applied.

^c^Log transformed data.

^d^Untransformed data.

Figures 5 and 6 show average daily colony growth of the respective pathogens, *Curvularia* and *Pestalotiopsis* species. At day one, there was a significant reduction in growth for both pathogens compared to the growth on control plate but there was slight difference in growth in the subsequent days for treated plates, control and Carbendazim amended plates.

### Effect of wood vinegar of spore production and spore germination

**Figure 3:**
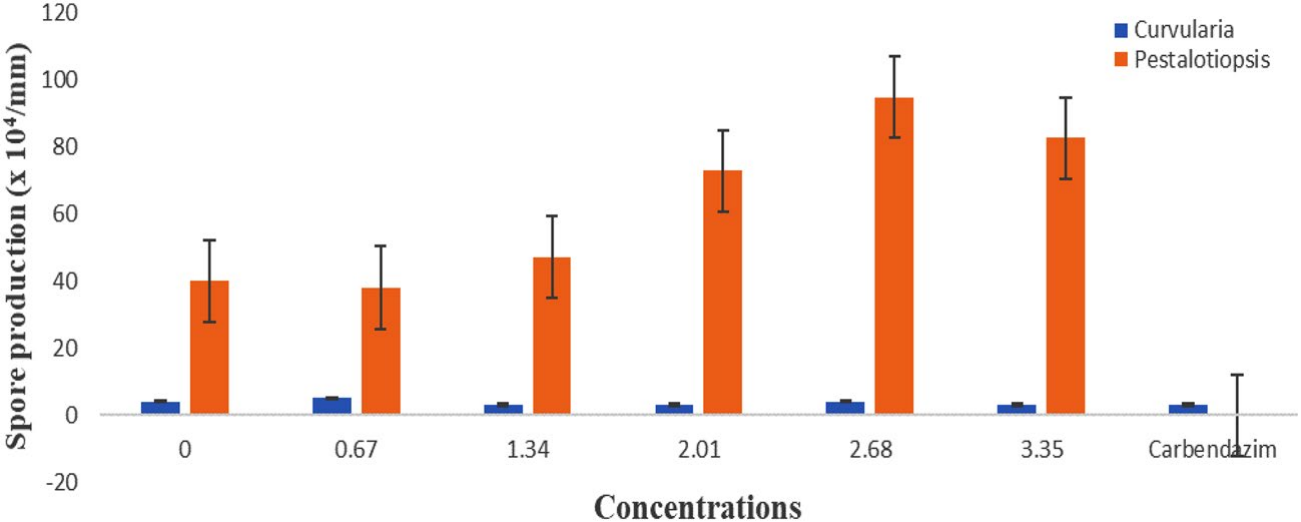
Effect of wood vinegar amended concentrations and carbendazim on spore production of the pathogens.

**Figure 4:**
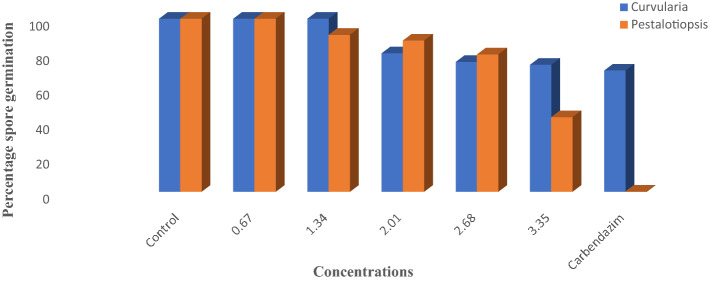
Effect of wood vinegar amended concentrations and carbendazim on spore germination of the pathogens.

Wood vinegar had minimal impact on spore production and germination at the highest concentrations (2.68 and 3.35% v/v) (Figs. 3, 4) for *Pestalotiopsis* species but generally caused very low spore production of *Curvularia* species (Fig. 3). The inhibition activity of wood vinegar on the developmental stages of the pathogens was minimal at the two lowest concentrations as compared to the highest concentrations above (Figs. 3, 4).

Table 2 shows effect of the concentrations on sclerotia production by *Curvularia* species at different days. Sclerotia produced on carbendazim treated plates were the highest followed by 2.01% v/v to 3.35%v/v of wood vinegar treated plates at day 13. Subsequently, at day 7 there was no observation of sclerotia on any of the treated plates including control plates. Generally, majority of the healthy wounded leaves inoculated with the two pathogens produced similar symptoms observed at the nursery.

**Table 2:** Sclerotia production of *Curvularia* isolate at selected days on different concentrations of organic farming aid and carbendazim.

Treatments (% v/v)	Presence of sclerotia		
	Day 7	Day 10	Day 13
0 (Control)	−	+	+
0.67	−	+	+
1.34	−	+	+
2.01	−	+	++
2.68	−	+	++
3.35	−	+	++
^2^Carbendazim (0.1)	−	++	+++

−Absent.

+Present but low.

++Medium.

+++High.

## Discussion

**Figure 5:**
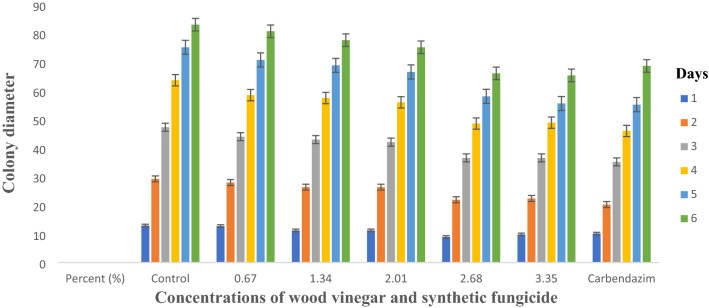
Average daily growth rate of *Curvularia* species on different concentrations of wood vinegar and Carbendazim fungicide.

**Figure 6:**
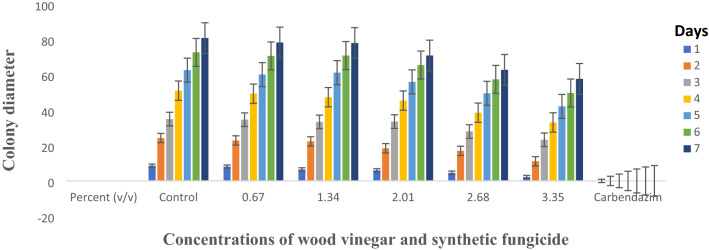
Average daily growth rate of *Pestalotiopsis* species on different concentrations of wood vinegar and Carbendazim fungicide.

Wood vinegar, commonly called wood acid, pyroligneous acid is known to contain several organic compounds including acetic acid, acetone, methanol, phenols and ketones which are produced from agricultural, forestry residue samples, bamboo, sawdust, cotton stalk, Chinese fir sawdust, et cetera^14–16^. Wood vinegars possess antimicrobial properties and suppress the growth of microbes at higher concentrations. The effectiveness of wood vinegar depends on several factors such as kind of wood used, level of temperature it is subjected to produce the resultant products^15^. Oramahi et al.^15^ observed growth inhibition of decay fungi at 0.5 to 1.5% (v/v). A similar result was achieved in the present study when concentration of wood vinegar was increased from 0.67 to 3.35% (v/v), both *Pestalotiopsis* and *Curvularia* species were inhibited at ≥ 20% for the highest concentrations (2.01 to 3.35% (v/v). A concentration was considered to be inhibitory when the inhibition percentage was ≥ 20% (Table 1, Figs. 1, 2). Bengyella et al.^16^ reported that *Curvularia* species sporulates profusely under harsh condition of high temperatures in or on putative hosts and conidia are easily dispersed in air, an observation made in this research work was production of numerous sclerotia (Table 2). Initially, growth of both pathogens were slow as compared to unamended plate but in the subsequent days, growth rates were somehow similar for treated plates and control plates with slight growth differences (Figs. 5, 6). Arango et al.^17^ reported that brown rot fungi including *F. palustris* secretes oxalic acid and possesses resistance against copper based wood preservatives whilst white rot fungi are more sensitive to natural chemicals extracted from white spruce (*Picea* *glauca*), Jack pine (*Pinus banksiana*), and red pine (*Pinus resinosa*) cones such as pinosylvin dimethyl ether and the extractives inhibited the growth of white rot fungi (*T. versicolor* and *Phanerochaete chrysosporium*), but slightly stimulated the growth of brown rot fungi (*Neolentinus lepideus*, *Gloeophyllum trabeum* and *Postia placenta*) at the 1:1:1 mixture of these compounds^18^. Oramahi and Yoshimura^19^ reported that wood vinegar from *Vitex* *pubescens* Vahl inhibited white rot fungus, *T*. *versicolor* and a brown rot fungus, *F*. *palustris*.

The phenol content of wood vinegar is likely responsible for its antifungal activity^15,20^. These results are at par with our findings in the present study. The antifungal action of wood vinegar might be due to its effect on fungal enzymatic activity. Kang et al.^21^ observed significant growth inhibition by some organic acids such as acetic, malic, oxalic and citric acid, with acetic acid causing the highest inhibition of fungal growth. Kang et al.^21^ examined *Colletotrichum gloeosporioides*, *C. coccodes* and *C. dematium* and confirmed an inhibition of respiration by acetic acid as measured in the culture media. Amendment of medium with acetic acid affected glucose utilization but glucose was re-used after the elimination of acetic acid, accompanying with the fungal growth^22^. Kang et al.^21^ however, observed increased catalase activity in *C. gloeosporioides* when hydrogen peroxide was added to the potato dextrose broth medium. But when hydrogen peroxide together with acetic acid was added to the medium, the enzyme activity rather declined slowly with incubation time, inferring that the interruption in enzyme induction might be related to an inhibition of respiration caused by acetic acid resulting in obstruction of energy metabolism.

According to Lekete et al.^23^, Carbendazim is an effective chemical against *Pestalotiopsis* species in vitro and is at par with our present finding when compared to wood vinegar (OFA). However, considering its inhibition effect, wood vinegar (OFA) is a promising product with fungistatic properties.

## Conclusion

Wood vinegar possesses antifungal properties which inhibited fungi growth at high concentrations due to the acids, phenol and other organic compound in its composition. High levels of wood vinegar concentrations inhibited both *Pestalotiopsis* and *Curvularia* species in-vitro.

## Methods

### Experimental site

The experiment was conducted at Council of Scientific and Industrial Research—Oil Palm Research Institute (OPRI), Plant Pathology Laboratory, Kusi. Kusi is located in the Dekyeambour District in the Eastern Region of Ghana. The rainfall pattern in Kusi is bimodal with an annual rainfall of 1621.2 mm and temperature ranging from 28.9 to 35.4 °C (Oil Palm Research Institute Meteorological Weather Station, 2020).

### Collection of diseased samples, isolation and purification of the pathogens

Symptomatic disease samples were collected from the OPRI nursery, kept in tablet envelopes, labeled and brought to the laboratory. Diseased leaves were washed under running tap water for one minute, they were surface sterilized in 10% (v/v) sodium hypochlorite solution and washed in two changes of sterile distilled water. They were blotted dry and plated on chloramphenicol amended water agar. Emerging fungal growth from peripheries of cultured leaves was sub-cultured onto a half strength cPDA and pure cultures were established for purification purposes.

### Pathogenicity test

Ten leaves from healthy oil palm seedlings were collected from the OPRI nursery, washed under running tap water, surface sterilized with 70% (v/v) ethanol, washed in two changes of sterile distilled water. The leaves were wounded with an inoculating needle and inoculated with the *Curvularia* isolate (KOP-21-5) and *Pestalotiopsis* isolate (KOP-21-20) separately. Five leaves were inoculated with each pathogen to prove Koch’s postulates^23^ in a humidified petri dish in the laboratory.

### Media preparation and fungicide amendments

Two percent (w/v) of 250 ml water agar was prepared and amended with 125 mg of chloramphenicol for isolation of disease pathogens. 39 g of Oxoid potato dextrose agar (PDA) was weighed (using Mettler PM 600 electronic balance) into a medium bottle, one litre distilled water was added for the bioassay of the pathogens. 100 ml of PDA was measured separately into seven 250 ml Erlenmeyer flasks and sterilized at 121 °C, 15 psi for 15 min in a Tuttnauer Autoclave. Upon cooling, the medium was adjusted to the required volume and amended with different concentrations of wood vinegar (Control (0), 0.67%, 1.34%, 2.01%, 2.68% and 3.35% v/v) and the manufacturer’s recommended rate of carbendazim (0.1% v/v).

### In vitro assay of foliar fertilizer against the pathogens

Food poisoning technique^24^ was employed to screen the organic product against pathogens. Five mm of actively growing mycelial plug of a seven-day old *Curvularia* species (KOP-21-5) and *Pestalotiopsis* species (KOP-21-20) were centrally placed inversely on each plate medium (five plates per concentration) and incubated at 31 ± 1 °C in a humidified transparent container under diffused sunlight during the day and darkness during the night (Five plates per treatment). Colony diameters were measured daily from the reverse side of plates with a ruler (average of two diagonal measurements per plate). Sclerotia production was scored qualitatively from day 7 to day 14 after incubation. Percentage inhibition was calculated using the formula by Chaurasia et al*.*^25^ expressed as:

$${\text{I}} = \frac{{{\text{C}} - {\text{T}}}}{{\text{C}}} \times 100$$

where I Inhibition percentage, C Average growth of control plate, T Average growth of fungicide treated plate.

Also, average daily growth rate was calculated with the formula below;

$${\text{Average}}\;{\text{growth}}\;{\text{rate}} = \frac{{\sum \, (A \, - \, B){\text{ for}}\;{\text{incubation}}\;{\text{period}}}}{{{\text{Total}}\;{\text{number}}\;{\text{of}}\;{\text{incubation}}\;{\text{period}}}}$$

A Average colony diameter for current day, B Average colony diameter for previous day.

### Effect of wood vinegar on different developmental stages of Curvularia and Pestalotiopsis species

After colony diameters were measured for the respective pathogens, six mycelial plugs (5 mm) of *Curvularia* species per plate (three plates per concentration) were excised along a radius centrally to the periphery of the culture^26^. Acervuli produced by *Pestalotiopsis* species after 10 days of culturing were harvested from three plates per concentration into 2 ml of sterilized distilled water in Bijoux bottle. The mycelial plugs of *Curvularia* were teased with a sterilized inoculating needle and shaken for two minutes on a Heidolph REAX 2000 vortex. 20 µl of spores were pipetted onto Weber Scientific International haemocytometer to determine spore count of both pathogens. Spore production was expressed as the number of spores per mm^2^ of colony area.

**Figure 7:**
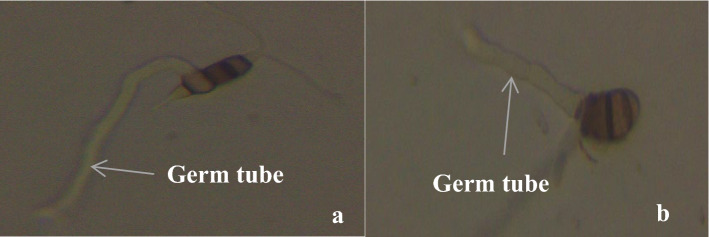
A germinating conidium of *Pestalotiopsis* species (**a**) and *Curvularia* species (**b**).

Spore germination was determined using a Riddell mount^27^. Three agar blocks per concentration (0, 0 0.67, 1.34, 2.01, 2.68, 3.35 and 0.1 Carbendazim) were used. 60 µl of spore suspension prepared from unamended PDA plates were spread at different locations on each setup. Germination of spore was measured after incubating the setup for 6 h at room temperature in the dark by examining 30 and 100 spores for *Curvularia* and *Pestalotiopsis* species, respectively at 100X magnification of an Amscope Compound Microscope (T490B). A spore was scored as germinated if the germ tube had reached at least the full length of the spore (Fig. 7).

### Experimental design

Completely Randomized Design (CRD) was used for the in-vitro assay. Both transformed (log transformation) and untransformed data were analyzed using GenStat statistical package 12th edition and the means were compared with Fisher’s protected Highest Significant Differences at 1% (Supplementary information 1).

### Research involving plants

Permission was granted before the use of oil palm seedlings and they were handled in conformation to institutional and national laws.

## Supplementary Information

Supplementary Information.

## Data Availability

The datasets used and/or analysed during the current study are available from the corresponding author on reasonable request.
