# Author Correction: Bioactivity of an organic farming aid with possible fungistatic properties against some oil palm seedling foliar pathogens

**DOI:** 10.1038/s41598-023-30022-2

**Published:** 2023-02-20

**Authors:** Joshua Obeng, Daniel Agyei-Dwarko, Peter Teinor, Isaac Danso, Hanif Lutuf, Emmanuellah Lekete-Lawson, Fred Kormla Ablormeti, Mary Akpe Eddy-Doh

**Affiliations:** 1 Council for Scientific and Industrial Research – Oil Palm Research Institute, P.O. Box 74, Kade, Ghana; 2 HJA Africa Limited, Accra, Ghana

Correction to: *Scientific Reports* 10.1038/s41598-023-27972-y, published online 23 January 2023

The original version of this Article contained an error in the Discussion section.

“A similar result was achieved in the present study when concentration of wood vinegar was increased from 0.67 to 3.34% (v/v), both *Pestalotiopsis* and *Curvularia* species were inhibited at ≥ 20% for the highest concentrations (2.01 and 3.34% (v/v).”

now reads:

“A similar result was achieved in the present study when concentration of wood vinegar was increased from 0.67 to 3.35% (v/v), both *Pestalotiopsis* and *Curvularia* species were inhibited at ≥ 20% for the highest concentrations (2.01 to 3.35% (v/v).”

The original Article has been corrected.

